# Frontal P300 asymmetry and congruence judgment: Retroactive switching is impaired during school day mornings in female adolescents^☆^

**DOI:** 10.1016/j.crneur.2024.100128

**Published:** 2024-03-27

**Authors:** Gabriel E. Byczynski, Amedeo D'Angiulli

**Affiliations:** a Trinity College Institute for Neuroscience, School of Psychology, Trinity College Dublin, Dublin, D02 PN40, Ireland; b Neuroscience of Cognition and Imagination and Emotion Research Lab, Department of Neuroscience, Carleton University, Ottawa, ON, K1S 5B7, Canada; c Children’s Hospital of Eastern Ontario Research Institute, Ottawa, ON, K1H 8L1, Canada

**Keywords:** Frontal asymmetry, Adolescent cognition, Stroop-like conflict, ERP, P300, Cognitive ability, School performance, Neuroeducation

## Abstract

Investigating frontal EEG asymmetry as a possible biomarker of cognitive control abilities is especially important in ecological contexts such as school and work. We used a novel approach combining judgment performance and hemispheric frontal event-related potential (ERP) P300 asymmetry (fP3As) to evaluate aspects of cognitive control (i.e., repetition and switching) in adolescent females over a two-week ordinary school period. While undergoing electroencephalographic recording, students performed a word-colour “Stroop-like” congruence judgment task during morning and afternoon sessions, on Mondays and Wednesdays. Proportion of incongruence and congruence trials was 75% and 25%, respectively. ERP analysis revealed larger “novelty” right hemispheric fP3As amplitude for infrequent congruence but equivalent or significantly smaller than left hemispheric fP3As amplitude for frequent incongruence. RTs increased with extent of right fP3As shift. Behaviorally, repeat trial pairs (i.e., congruent followed by congruent, incongruent followed by incongruent) generally did not differ by time or day and were associated with near-ceiling accuracy. In contrast, switch trial pairs (i.e., congruent followed by incongruent, incongruent followed by congruent) in the afternoon were slower and associated with lower accuracy at the expected 75% criterion rate (i.e., judging incongruence by default), dropping significantly below 75% in the mornings. Crucially, compared to afternoon, morning fP3As patterns did not change adaptively with switch trial pairs. Although retroactive switching during congruence judgment was affected at all testing times, we conclude it was most impaired in the mornings of both early and mid school weeks, supporting misalignment between adolescent circadian cycle and school start time. We discuss some implications for optimal learning of adolescents at school.

## Highlights

• P300 amplitude asymmetry correlates with reaction time, possibly representing cognitive control.
• Frontal P300 amplitude asymmetry represents a plausible correlate of cognitive control via task switching.
• Markers of cognitive control vary with time and day of testing and is reflected in behavioural changes.

## Graphical abstract

**Figure:**
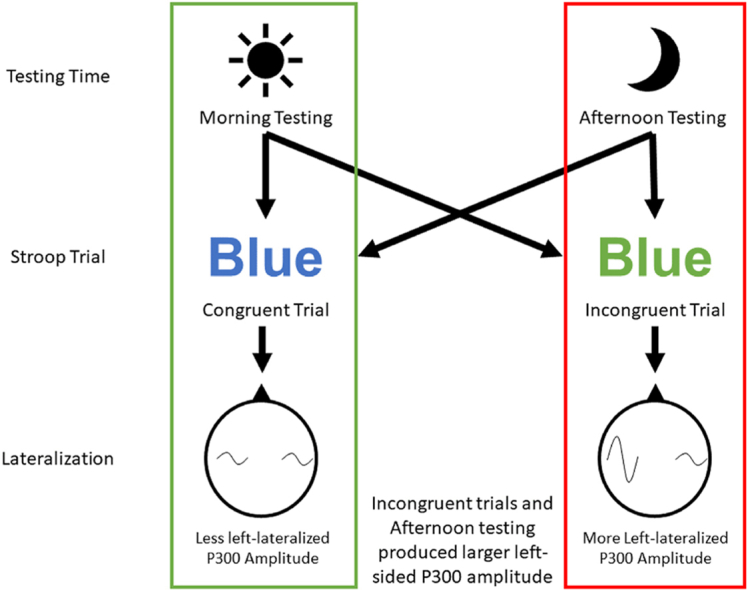

## 1 Introduction

A growing body of literature in cognitive and educational neuroscience research suggests that correlates of cognitive control can be reliably measured through EEG activity asymmetry differences between the left and right frontal regions (Ambrosini and Vallesi, 2016; Coan and John, 2003; Pérez-Edgar et al., 2013). Increasing consideration of frontal EEG asymmetry as a possible biomarker of neurocognitive functions seems especially important in everyday ecological settings such as school and work (Kirby and D'Angiulli, 2009). However, current findings are also mixed and unclear (Taillard et al., 2021). We used a novel approach leveraging converging evidence from cognitive performance, hemispheric event-related potential (ERP) asymmetry, and selective probing of the frontal P300 signature (P3a, Squires et al., 1975) to gain insights on aspects of cognitive control (i.e., proactive and reactive) in adolescent female students during a word-colour Stroop-like judgment task, and to examine if and how this high-level neurocognitive aspects may be affected by circadian variations (morning vs afternoon). Our ultimate motivation is to contribute novel neuroscientific evidence to inform best educational practice.

### 1.1 Event-related potentials, applications to educational neuroscience

To inform important aspects of student learning –such as school starting times (SSTs), testing times, sleep/nap duration guidelines, and break duration – their impact on cognition, and particularly, which relevant and specific domain of cognition, must be well-understood. It is known that testing performance declines over the school day, thus creating justification, or at least consideration, for a potential standardized testing time to reduce variability, and to optimize student performance (Sievertsen et al., 2016). In one example, a 20-min alteration in sleep duration in children was shown to produce objective attentional and emotional differences, highlighting an important aspect to consider due to clear influences on the attentional domain of cognition (Davidson et al., 2022). One well-studied factor is the effect of the time-of-day on cognition. Studies have found that SST and sleep duration are influential factors in academic performance, particularly regarding attention, learning, and memory consolidation. Indeed, delaying SSTs benefits student academic performance (Alfonsi et al., 2020).

While many studies quantify the effect of SST and time-of-day using standardized measures such as testing scores or the psychomotor vigilance task (PVT) with good success, there is also a breadth of research that provides context to the target behaviors with neural correlates of cognition. For example, frontal N2 and frontal P300 effects are reliable ERP markers of inhibitory control in children (Linnavalli et al., 2022, Pietto et al., 2018), and intervention can beneficially influence this marker (Pietto et al., 2018). Furthermore, frontal asymmetry (an uneven hemispheric activation) has been shown to correlate with numerous cognitive domains involving effortful control including attention (Longarzo et al., 2020), mental imagery (D'Angiulli et al., 2021), decision making (Neal and Gable, 2019), and auditory executive functions (Byczynski et al., 2022).

### 1.2 P300 and cognitive control

Historically, the most widely used ERP marker is the P300 family of waveforms (Andreassi, 2010; Luck, 2014). Two of the most reliable and consistent are known as the relatively more posterior (parietal) P3b and relatively more anterior (frontal) P3a (Squires et al., 1975), with the first component being implicated in updating the expectation contingent target probability during selective attention and the latter being involved in allocation of selective attention. The most influential theoretical framework which integrates the P3a and P3b components has been proposed by Polich (2007). On that perspective, the role of the P300 is primarily in context of attentional resource allocation and cognitive demand. Specific to P300 amplitude, we here focus on two major aspects of Polich's framework, the so-called context-updating theory (Donchin and Coles, 1988), and attentional shift as resource allocation (Gumenyuk et al., 2004). The former theory implicates an attentionally modulated process whereby a comparison mechanism is used between subsequent stimuli. Thus, when presented with relatively infrequent (i.e., “novel”) stimuli, the current neural representation must be updated, eliciting a larger frontal P300 component than when the stimulus is repeated, and internal representation is validated (see also Fig. 2 (Polich, 2007),). The second concept, resource allocation (Kahneman, 1973) suggests that there exists a relationship between demand of attentional resource allocation and P300. When tasks are “easy”, eliciting minimal attention, P300 amplitude is said to be larger, while more demanding tasks elicit greater attentional resource, and smaller amplitude. As detailed later, a further development of this model (Polich, 2012) links the trade off effects of novelty vs demand on the P300 amplitude to the often-observed hemispheric asymmetric activity shown by this ERP signature, grounding it on the underlying neural substrate of attentional pathways.

Most recently the P300-like or P300 family has been linked with cognitive control (e.g. (Enriquez-Geppert and Barceló, 2018),). One definition of cognitive control in ERP research has been: *the ability to utilize an internal representation to flexibly adapt to changing environments* (J. Wu et al., 2022). To probe this aspect, tasks like the ‘task-switching paradigm’ have been used, in which repetitive trials are contrasted with switch trials and more flexible ability to adjust for changing task goals is viewed as more efficient. Two underlying mechanisms have been proposed: task-set inertia and the task-set reconfiguration (Wu et al., 2022). The former model suggests that speed and accuracy costs arise from the interference between subsequent tasks, while the latter model posits costs originate from endogenous top-down processes, indicating need for corrective additional cognitive control and effort. Accordingly, cognitive control in task switching might be explained by both inhibition and task-set reconfiguration (Vandierendonck et al., 2010). In task switching, and because of changing cognitive demands, it has been shown that P300 complex in the frontal regions represent a novelty effect, suggesting that *graded* modulation of positive amplitude in the frontoparietal network represents the handling of changing cognitive demands.

Switch costs have been traditionally studied in the context of Stroop and Strop-like^1^ experimental paradigms. Wu et al. (2015) show that flexibility in alternating internal goals (i.e., cognitive control) evoked frontal asymmetry, in which switching effects manifested over the frontal right region (which the authors attribute to inhibitory control). This inhibitory control was said to be involved in the control required during switching, consistent with the task set inertia (S. Wu et al., 2015)). Thus, the current theory suggests that as a measure of cognitive control (flexibility) through task switching, deficits in cognitive control can be inferred by strength of evoked response to task switching. It is known that the magnitude of the effect of conflict interference can be modulated by manipulating the proportion congruency (PC), that is, the relative frequency/likelihood of congruent trials within the task blocks (Gonthier et al., 2016; Logan and Zbrodoff, 1979). The basic assumption is that information about the PC is used to adjust the cognitive control level and, as the size of the Stroop inversely reflects the success of interference resolution, it is postulated that when such effect is relatively small, a greater extent of cognitive control has been recruited (e.g., Braem et al., 2019; Lindsay and Jacoby, 1994). Specifically, when incongruent trials are more frequent (low PC), the high probability of encountering incongruent trials and experiencing interference increases cognitive control demands and this yields significantly smaller Stroop effects (i.e., reduction of interference). Conversely, when congruent trials are more frequent (high PC), due to lower frequency of incongruent trials, cognitive control is more relaxed and the interference increases (e.g., Lindsay and Jacoby, 1994).

A generalized model of response repetition and switching is the influential theory of Dual Mechanisms of Control model (DMC, Braver, 2012; Zurrón et al., 2014). The DMC explains cognitive control in terms of coordination of two distinct types of cognitive attitudes: proactive and reactive. The former operates by maintaining actively a representation of the task goals and anticipatorily biasing cognitive processes according to task demand. Thus, proactive control acts as a predictive mechanism, engaged in a sustained fashion even before cognitively taxing events, like incongruences, occur. When proactive control is active, interference is reduced because top-down attentional biases select task-relevant information. Whereas reactive control is engaged transiently only on a need-basis before the point of no return, as such, it relies upon a recalibration mechanism reflecting the bottom-up reactivation of task representation and goals to adapt to interference.

Literature which discusses *reactive switching* suggests that sub-genres of cognitive control, such as reactive control, govern the retrieval of associations based on task demands (Kang and Chiu, 2021). Similarly, when preparing to engage in a task, goal-relevant information can be selected prior, such as in *proactive repetition*, whereby processes such as proactive (cognitive) control maintain goal-relevant selection decided prior to the task. Thus, there may be a give-and-take between these processes, in which a proactive control approach may be prominent, until task switching activation, resulting in a transient activation of regulative corrections (Mäki-Marttunen et al., 2019). In the context of Stroop-like tasks, where congruent and incongruent trial types occur with different frequency (PC), one might vary either cognitive control process. Accordingly, depending on which is the most frequent type of trial, proactive control governing (proactive) repetition would naturally settle as the default approach (i.e., if 75% incongruent, then 75% accuracy would indicate a constant and stable proactive mode). Indeed, if reactive control becomes the dominant mode when the task switches, accuracy would increase, based on ability to flexibly adapt the internal goals by shifting between reactive and proactive control (i.e., either switching or repeating). Thus, coupled with changes in P300, variations in RTs and accuracy which are dependent on trial repetition versus trial switching may be a possible tool to probe cognitive control.

### 1.3 Frontal P300 asymmetry (fP3As)

Frontal EEG and ERP asymmetry (henceforth *fP3As*) has been used specifically in relation to academic performance, but the findings are varied. In one study, frontal alpha EEG asymmetries were found to correlate with how students cope with examinations (Papousek et al., 2019), and in another study it was found that P300 latency asymmetry was associated with behavioural disturbances (including academic performance-see Gerez et al., 1999). However, to emphasize the variability of results pertaining to asymmetry and cognitive ability, there are studies that reported asymmetry to be associated with specialized brain function, for example decision making (Cunningham et al., 2005), while others found it unrelated to cognitive features at all (Kutas et al., 1988). Beyond behavioural correlates of asymmetry, it has also been shown that frontal EEG asymmetry score varies in relation to the seasonal and chronological instance of EEG recording (Peterson and Harmon-Jones, 2009). Indeed, it was found that circadian and seasonal variability (through cortisol levels and mood changes) influence resting asymmetrical frontal activity (Peterson and Harmon-Jones, 2009). Specifically, in that study right frontal activity was relatively greater in the mornings, possibly as a result of the duration of time awake before recording (Velo et al., 2012).

In educational research, ERP asymmetry is most often used in the context of reading, with studies implicating ERP asymmetry as a correlate of phonological awareness (Norton et al., 2021), a predictor of reading skill (Segalowitz et al., 1992), and even school grade (Licht et al., 1992). Its use in educational contexts makes ERP asymmetry a suitable target when investigating factors affecting school performance, including SSTs and test timing. While many studies investigate ERP asymmetry as a correlate or predictor of academic performance, there are few studies to our knowledge which use this measure in the context of the chronobiological influence on cognitive processes. There is evidence that suggests that P300 amplitude changes as a result of time of day, with some studies attributing its change to food-intake (Geisler and John, 1992), or circadian variation in resource recruitment (Wesensten et al., 1990). Therefore, an important target for neuroeducational research which still needs to be properly assessed is the coupling of cognitive performance and chronological contexts of ERP asymmetry.

Since right-hemispheric signal activation has been shown to correspond to executive control of response, with faster response times (RTs) during an inhibition control task (Garavan et al., 1999), it is possible that a selective ERP signature such as the frontal P300 may reliably reflect hemispheric correlates of resource recruitment related to evaluating and responding to conflicting information. The P300 amplitude has been implicated in inhibitory ability, with one study finding a correlation between reduced P300 amplitudes and decreased Go/NoGo accuracy, in persons with Mild Cognitive Impairment (López et al., 2016). Furthermore, task switching literature has also reported changes in the P300 amplitude as a biomarker of working memory load and task demand (Wu et al., 2022). The P300 component has also been shown to reflect detection of task-relevant stimuli, and regional neural inhibition, while asymmetrical processes are believed to play a number of roles, including preventing inter-hemispheric conflict, or allowing for simultaneous complementary processes to co-occur (Hirnstein et al., 2008; Schofield et al., 2009) and, pertinently, previous findings combining EEG/ERP and fINS show lateralized functional connectivity increases during incongruent Stroop task trials (Chen et al., 2023).

Consistent with the reviewed evidence, it is possible that the aspects of cognitive control involving resource recruitment and attentional allocation can be measured through changes in right fP3As and might reflect, at the level of underlying brain processes, *neuroinhibition* (Polich, 2012). According to this framework, P300 could reflect rapid inhibition of neural activity to facilitate the transmission of information from frontal (P3a) to temporal-parietal (P3b) locations. P300 signals may originate from the initial need to enhance focal attention to isolate the task-relevant contents of working memory during stimulus detection (cf. (Klimesch et al., 2007). Reduction of extraneous neural noise would focus incoming stimulus information and sharpen memory encoding. When focal attention for the mot frequent stimulus is disrupted by the detection of an infrequent distracter or a target (stimuli that capture attention automatically or purposefully from task demands), the P3a is generated by the change in frontal lobe activation pattern (Bledowski et al., 2004). Under these conditions, converging neurophysiological, cognitive, ERP and fMRI data indicates greater activation for the right compared to the left hemisphere. This higher right-hemisphere activity is in line with the established evidence for the frontalparietal right-hemisphere attentional network system (Posner and Dehaene, 1994) and that traditionally larger P300 amplitude is found over the right compared to the left frontal/central areas in oddball tasks (Alexander et al., 1995, 1996).

### 1.4 The present study

The misalignment between school start times and adolescent circadian rhythms is already cause for concern, as sleep deprivation increases risk of mental health disorders, substance abuse, and academic performance (Wahlstrom, 2020), female adolescent students are disproportionately more at risk as compared to male counterparts (Agathão et al., 2020). Using a Stroop-like paradigm, in our previous study (D’Angiulli et al., 2021), we analyzed correlations between RTs, accuracy, time of day, day of week, EEG data, and sleep log data to explore relationships between these variables in a sample of female adolescent students. We found converging evidence that a 2-h social jet lag (SJL) during the week end as well as mental fatigue associated with sleep homeostatic pressure during the afternoon of midweek school days were correlated with cognitive declines in performance and changes in midline P300. A limitation of that study, however, was that we did not pinpoint what specific aspect of cognitive performance was impaired in association with the observed chronobiological interactions.

In this follow up study, we re-examined those neurocognitive data by taking a completely different and novel approach. Based on our previous findings of circadian misalignments reflected by time-of-day (morning vs afternoon) effects, we expected that morning as opposed to afternoon testing would produce a selective impairment of reactive control involving switching and reconfiguration of the task-set built in the design of our word-color congruence judgment task. Specifically, our key re-analysis considered successive pairs of congruent and incongruent trials to understand how behavioural data (i.e., reaction time and accuracy) may differ at the item-specific level. We determined the mean reaction time and accuracy for four conditions pairs of trials: congruent following congruent (*cC*), congruent following incongruent (*iC*), incongruent following incongruent (*iI*), and incongruent following congruent (*cI*). The rationale is that RTs and accuracy of congruent and incongruent trials can be probed in the context of the preceding trial (as done in conflict adaptation ERP research, see (Larson et al., 2014; Larson et al., 2009).

Specifically, because in our experiment the incongruent trials were three times more frequent than congruent trials (i.e., PC = 25 %), we expected the students to perform at near-ceiling levels in both morning and afternoon by coordinating proactive strategy for the more frequent incongruence judgement (repeating the semantic processing of the name of color) but by retroactively switching (judging the color of the stimulus) for the infrequent but easier congruent trials, when the same types of trials (cC or iI) occurred. Outcomes driven by proactive control are generally known as list-level congruence probability (LLCP) effects. The iI trials therefore should reflect mainly proactive repetition whereas cC trials should reflect the combination of proactive repetition and reactive switching. As demonstrated by scores of studies on cognitive control using similar low PC design (see review in (Viviani et al., 2023), also (Hutchison et al., 2010; S. Wu et al., 2015)) for this type of repeat paired sequences RTs should show *priming* (Puccioni and Vallesi, 2012) and low or no accuracy *costs* (Hommel, 2004), demonstrating effective reduction of the overall interference and of the difference between easy and difficult trials. Consequently, one should expect fP3As to show a large novelty right asymmetry amplitude for the infrequent congruent trials, whereas it should show a depressed, “higher load” amplitude for the frequent incongruent trials, namely, equal, or less P300 amplitude compared to that on the left hemisphere.

However, for pairs of “switch” sequences that did not feature the same type of trials (cI or iC), we hypothesized an increase in interference effect and a drop to less optimal accuracy performance in afternoon and, crucially, a significant impairment of performance in the morning because of sleep misalignment induced by school start times (D’Angiulli et al., 2021). These other types of interference effects are generally known as Item-specific congruency probability (ISCP) effects. The trials under this category should mainly reflect retroactive switching. We predicted that in the latter trials, impaired reactive switching would introduce speed and accuracy costs, particularly heavy in the mornings, and, concurrently, there should be no differential adaptive changes depending on congruence or incongruence trials in morning fP3As.

## 2 Materials and methods

### 2.1 Participants

Participants were recruited from a high school in Southwestern Canada (n = 24), with mean age of 16.9 years (SD = 0.4). Participants had European ancestry, and were subdivided as being middle-high socioeconomic status (Class I-II – Hollingshead Inventory, Bornstein et al., 2003). Participants received credits for participation, and signed consent was received from both student and one parent or guardian. All participants selected were females, as it has been shown that the effects of sleep deprivation are more prevalent in females, and furthermore using a female sample allows us to reduce sex-based variability (Becker et al., 2018; Lindberg et al., 1997; Putilov et al., 2021). All selected students had right hand preference.

For a more detailed methodology, please see D’Angiulli et al. (2021) of which this paper is an extended secondary analysis.

### 2.2 Ethics

This project was approved by the ethics committee of British Columbia's School District 73, Thompson Rivers University, and Carleton University in Accordance with the 1964 Declaration of Helsinki ethical standard, and the Tri-Council Policy Statement. (http://www.pre.ethics.gc.ca/pdf/eng/tcps2-2014/TCPS_2_FINAL_Web.pdf).

### 2.3 Word-color judgment task

Participants performed a Stroop-like *word-colour judgment* task, and were randomly assigned to two groups, the morning test group or the afternoon test group. The morning group was tested between 9:30–10:30 a.m., while the afternoon test group was tested at 12:15–2:15 p.m. Both groups were tested on Monday and Wednesday of each week at their designated time. Testing took place in a quiet space and involved a series of single trials in which participants assessed whether or not, on each trial, the word meaning matched the colour of the font it was written. For example, the word ‘RED’ could be shown, in blue font, and the participant would be required to respond with a left mouse click indicating that it was incongruent. If the word ‘RED’ was shown in red font, the participant would instead respond with a right mouse click indicating congruent. The response mouse was kept stationary through a Velcro pedestal base situated at the right of the desktop flat monitor screen. The choice of this paradigm allows for stimulus evaluation with a minimal motor response required which reduces motor ERP artifacts (see Zurrón et al., 2014).

The most important reason which led us to select this type of paradigm is that color-word congruency judgment, differently than standard versions of verbal Stroop, does not introduce confounds of stimulus-response contingencies because the response is not based on the association between a learned sequence between the two stimulus dimensions. In the present congruency judgment task, *both dimensions*, color and semantic meaning of the word, must be re-activated together, because the judgment hinges on their relationship, not their identity. This eliminates the effect of possible learned orders in the Stroop-like trials which may allow participants to implicitly learn the statistical structure of the sequence of trials and be able to respond accordingly, as argued by contingency explanations (e.g., Schmidt and Besner (2008)). Consequently, our task excludes by default predictions of correct response based on statistical inferences of contingency probability of the cuing dimension (such as, for example, basically relying on the frame of reference of alternating matching response provided by automatic word reading) because what is to be predicted in a higher level relationship of congruence not simply S–R (for a similar point see discussion in Bugg and Crump (2012).

Each word was presented for 200 ms, with relatively long inter-stimulus interval (1000 ms). Responses deadline was 1000 ms after stimulus presentation. Words were generated with Stim2 software and presented in the center of a 17” Dell LCD monitor (with the following settings: contrast ratio = 400:1, hue = 4000 K, and luminance = 120 cd/m2). The words were capitalized at 18-point size with Arial type font at approximately 2 degrees of visual angle (60 cm from chin rest). The order of presentation was randomized. Stimuli were presented at a 3:1 ratio of incongruent (n = 224) to congruent (n = 76) stimuli. The procedure followed an ‘Oddball Stroop’ task, which results in a high incongruency demand, and thus high task control requirement; a task design which has been used in many other studies (Melcher and Gruber, 2006; Milham et al., 2003; Rosenfeld and Skogsberg, 2006).

Reaction times (RTs) and accuracy were recorded for each trial. The mean accuracy (the mean percent of correct responses) was calculated for each time and day combination, and for each of the congruent and incongruent stimuli. Data were collected over a 2-week period and averaged across each consecutive weekday (Data collection specifics, e.g., time of year, daylight, and their effects were the focus of D’Angiulli et al. (2021) and are reported in detail there.).

### 2.4 Electroencephalography

EEG data were recorded with Neurosoft, Inc. EEG quick caps (AgCl electrodes; Sterling, USA). Each participant had nine electrodes, Pz, P3, P4, Cz, C3, C4, Fz, F3, and F4, placed according to the international 10–20 system. Electrodes were referenced to the average, and vertical electrooculograms were recorded at the left and right supraorbital and zygomatic regions. EEG recordings were amplified and digitized with a SynAmps2 and SCAN 4.3 EEG system (Compumedics Neuroscan, El Paso, USA). Data were filtered with high pass 0.15 Hz and low pass 100 Hz filters. Data were digitized at 1000 Hz sampling rate. EEG was discarded if there were bad channels during recording or the task was not completed (n = 4). Each participant's EEG was epoched from 100 ms pre-stimulus to 1000 ms post stimulus and averaged to the onset of each word presented. EEGs were baseline corrected using a 100ms pre-stimulus interval. EEG epochs were discarded if the EEG exceeded ±100 μV threshold (∼5%). Trials with incorrect responses were not further analyzed (∼20%). We focused our analysis in this paper on the F3 and F4 electrodes, as they correspond to regions commonly approached in regards to frontal asymmetry (Kelley et al., 2017; Zhao et al., 2018). P300 amplitudes were determined using a binning procedure in which the time points of interest were extracted by visual inspection of the P300 wave (e.g., time range from 250 to 400 ms). The highest positive value was considered the amplitude of the P300 event.

### 2.5 Data analysis

We first analyzed RTs by time of testing, day of testing, and categorized by trial type (i.e., Monday: AM-congruent, AM-incongruent, PM-congruent, PM-incongruent) using a two-sample *t*-test in order to determine how behavioural measures of cognition differed by time-of-testing. Next, we contrasted the F3- and F4–P300 amplitudes for the same categorization using a two-sample *t*-test. This was done in order to determine how fP3As varied during time and trial type. fP3As was determined via subtraction (F3–P300 amplitude – F4–P300 amplitude]). Contrasts were performed between both morning/afternoon testing and congruent/incongruent trials using two-sample t-tests. A 2-way ANOVA was run on time of day and day of week separately for the congruent and incongruent trials in order to determine if an interaction between time and day existed. Pearson correlation was performed between fP3As and reaction time for each time point and trial type to reveal behavioural correlations. A Bonferroni correction was applied for multiple comparisons. Significance was considered at *p* < 0.05.

Lastly, we performed a trial-level analysis examining successive pairs of congruent and incongruent trials to understand how behavioural data (i.e., RTs, accuracy) may differ in relation to repetition and switching. We determined the mean reaction time and accuracy for four conditions pairs (cC, iC, iI, cI), such that the RTs and accuracy of congruent and incongruent trials can be assessed in the context of the preceding trial (Larson et al., 2009). To determine if differences existed between congruent and incongruent trials, we preformed paired-sample t-tests (within day and time conditions), between “repeat” (cC and iI) and “switch” (iC and cI) trial pairs. Using these data, we compared accuracy to a theoretical 75% accuracy criterion via one-sample *t*-test, which would be the result if participants responded ‘incongruent’ to all trials, regardless of congruency. However, subdividing the ERP data according to the different trial types (cC, iI, cI, iC) unfortunately produced unbalanced cells with unreliable differences given the low statistical power, therefore, we adopted the alternative approach of drawing logical correspondences between the behavioral patterns and more global ERP congruent vs incongruent effects, that is, we used a so-called converging evidence approach (Algom and Fitousi, 2016) rather than a direct statistical correspondence between the behavioral and the neuro-electrophysiological measurements.

## 3 Results

### 3.1 Trial-based behavioural data

**Fig. 1:**
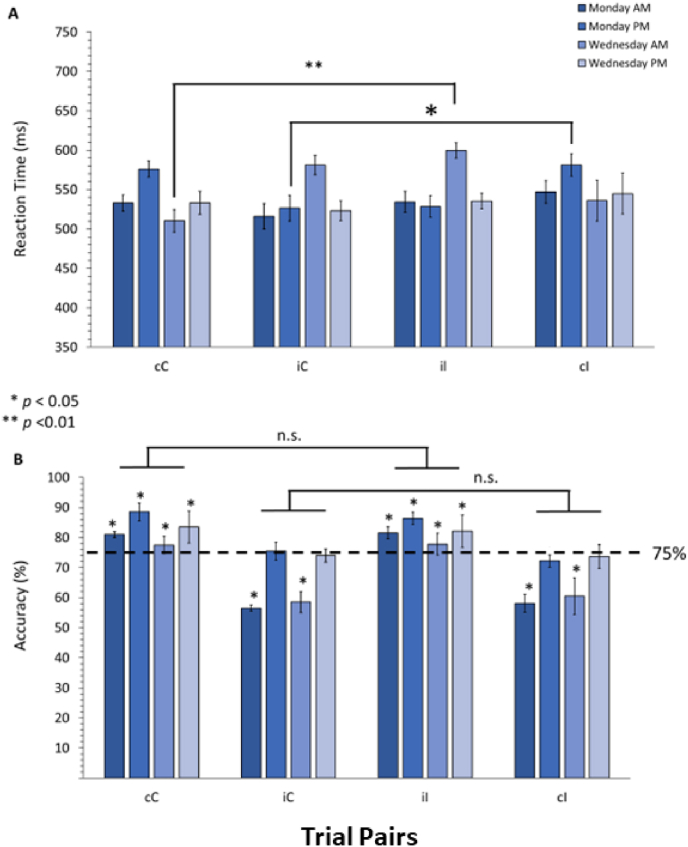
Behavioural data A) Reaction times and B) Accuracy for trial pairs for each day and time condition combination. Dashed line indicates 75% accuracy. * denotes significance (p < .05).

In order to investigate effects of proactive repetition and reactive switching, we examined how RTs and accuracy may differ by trial type. Mean RTs and accuracy for each trial pair are shown respectively in panel A and B of Fig. 1.

**Fig. 2:**
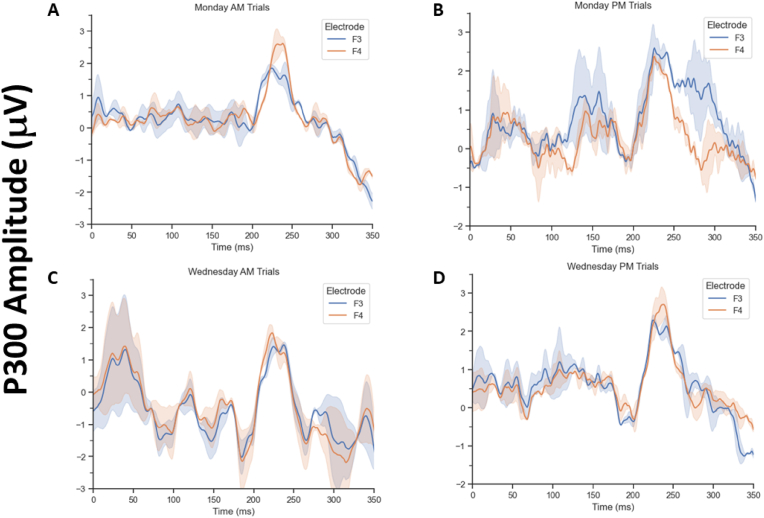
Event-related potentials recorded from electrodes F3 and F4 where P300 amplitude was significantly (p < .05) different between electrode pairs. A) Monday AM sessions F3 and F4 ERP averages of both congruent and incongruent trials. B) Monday PM C) Wednesday AM D) Wednesday PM.

Among all RT comparisons, only three significant differences were detected, following FDR correction (see Fig. 1A). On Monday afternoon, iI trial pairs had significantly faster RTs than cC pairs (*t*(23) = 2.80, *p* < .01). On Wednesday morning, iI trial pairs had significantly slower RTs compared to cC (*t*(23) = 5.11, *p* < .01), and on Monday afternoon, iC trial pairs had significantly faster RTs as compared to cI (*t*(23) = 2.53, *p* = .01).

For accuracy (see Fig. 1B), there was no significant difference between incongruent and congruent trials pairs (i.e., cC vs. iI, or iC vs. cI). Comparing accuracy to the expected 75% response criterion, when proactive repetition and reactive switching were selected, performance rate varied significantly at specific time and day conditions. All conditions (Mon/Wed and AM/PM), except Wednesday afternoon, had significantly higher accuracy during repetitions, that is, congruent trials preceded by congruent trials (cC), and similarly for incongruent trials preceded by incongruent ones (iI).

In contrast, almost all cI trial pairs, mainly reflecting retroactive switching had significantly lower accuracy than 75%. The only exception was on Wednesday afternoon, in which performance was no different than criterion. Finally, for iC trial pairs, again mainly reflecting reactive switching, morning testing on both Monday and Wednesday had significantly lower accuracy than 75% but were right at criterion level performance in the afternoon.

### 3.2 P300 amplitude

The P300 amplitudes, organized by electrode, time of day, day of testing, and trial type, are presented in Table 1. P300 amplitudes were significantly (*p* < 0.05) larger at the F4 electrode compared to F3 during both congruent and incongruent trials on Monday morning. On Monday afternoon, incongruent trials yielded significantly larger F3 P300 amplitudes compared to F4, whereas no asymmetry was observed for congruent trials. On Wednesday testing, for both morning and afternoon, congruent trials yielded a significantly larger P300 amplitude at the F4 electrode. However, there were no significant asymmetries for incongruent trials.

**Table 1:** P300 amplitudes for F3 and F4 electrodes, for each trial type, time of testing, and day of testing.

Day of Testing	Time of Testing	Trial Type	Electrode	P300 Amplitude (mV) (mean ± SD)	*t*-test (*t* and *p* values)*	Significant Asymmetry
Monday	AM	Congruent	F3	2.03 ± 0.41	*t(23)* = 6.78	Right
			F4	3.07 ± 0.63	***p* <** 0**.001**	
		Incongruent	F3	1.74 ± 0.36	*t(23)* = 5.34	Right
			F4	2.40 ± 0.49	***p* <** 0**.001**	
	PM	Congruent	F3	2.62 ± 0.53	*t(23)* = 3.89	None
			F4	2.44 ± 0.50	*p* = 0.935	
		Incongruent	F3	3.20 ± 0.65	*t(23)* = 4.33	Left
			F4	2.47 ± 0.50	***p* <** 0**.001**	
Wednesday	AM	Congruent	F3	1.69 ± 0.35	*t(23)* = 3.57	Right
			F4	2.09 ± 0.43	***p* = .003**	
		Incongruent	F3	1.55 ± 0.32	*t(23)* = 1.80	None
			F4	1.72 ± 0.35	*p* = 0.339	
	PM	Congruent	F3	2.43 ± 0.50	*t(23)* = 4.40	Right
			F4	3.16 ± 0.64	***p* <** 0**.001**	
		Incongruent	F3	2.16 ± 0.44	*t(23)* = 1.28	None
			F4	2.33 ± 0.48	*p* = 0.822	

*p-values are Bonferroni corrected.

ERP traces for each day and time combination are shown in Fig. 1 to illustrate this finding. (The specific values and statistical results are reported in Table 1). We illustrate the time of day and day of week effect on the ERPs averaged over incongruent and congruent trials collapsed together in Fig. 2 (see section 2.5 for rationale). Results presented here focus on central tendencies, therefore averages and standard deviations are shown to best illustrate the general group effects (individual differences will be the topic of a separate, in-progress study).

### 3.3 fP3As vs RTs

**Fig. 3:**
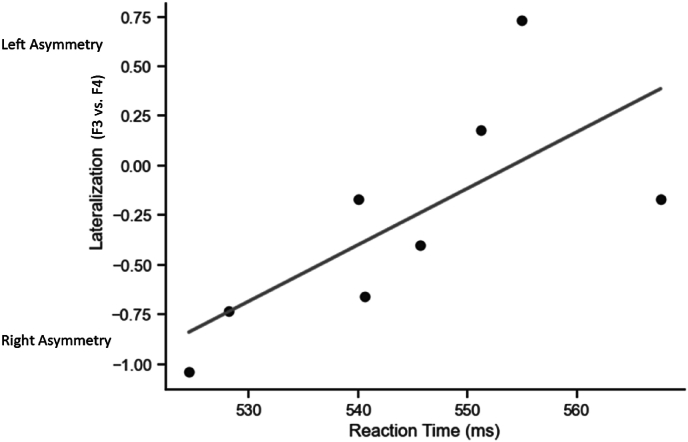
F3 vs. F4 frontal P300 amplitude asymmetry (measured as difference scores in microvolts) and reaction time (ms), across Monday and Wednesday, morning and afternoon testing, with regression line (in grey).

When we regressed RTs on the extent of fP3As expressed as difference between F3 and F4 electrode amplitudes, we found a significant positive correlation between Left-sided P300 amplitude and RTs (*r*(6) = 0.87, *p* = .026). This analysis is represented graphically in Fig. 3.

### 3.4 Frontal P300 asymmetry

**Fig. 4:**
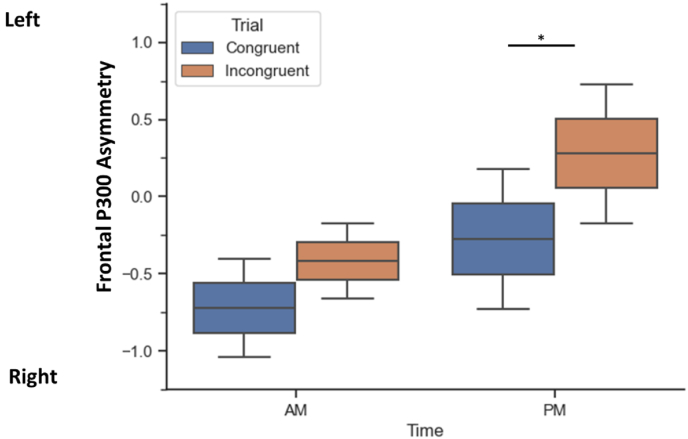
Mean frontal P300 amplitude asymmetry (F3 vs. F4) for time of testing showing significant increase in left asymmetry in the afternoon for incongruent trials. Error bars represent ±1 standard deviations. Asterix denotes significance at p < 0.05.

The results of an ANOVA analysis, graphically represented in Fig. 4, showed significant interactions between time of day and day of week for congruent trials (F(1,23) = 9.51, p < 0.01), incongruent trials (F(1,23) = 14.31, p < 0.001). When comparing F3–P300 amplitude asymmetry during each trial type using a post hoc analysis with (Bonferroni corrected) paired t-tests, we found no significant difference in asymmetry on Monday vs Wednesday mornings. There was a significantly larger asymmetry during incongruent trials on Monday and Wednesday afternoons (Monday PM: *t(23)* = 2.44, *p* = 0.018, Wednesday PM: *t(23)* = 2.64, *p* = 0.011).

**Fig. 5:**
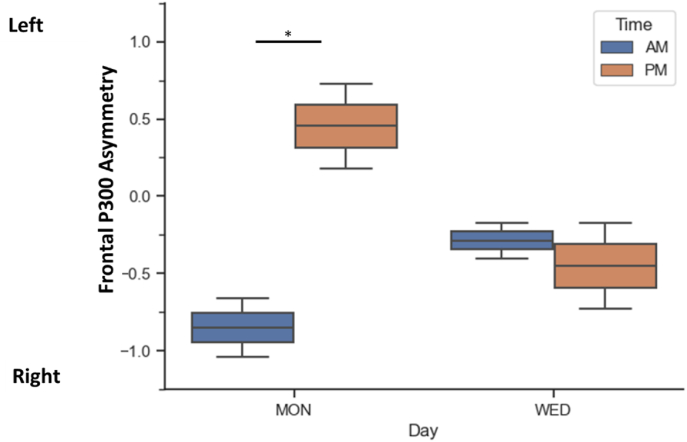
Mean frontal P300 amplitude asymmetry (F3 vs. F4) for day of testing showing significant increase in lateralization on Monday. Error bars represent ±1 standard deviations. Asterix denotes significance at p < 0.05. Y axis represents difference scores in microvolts.

Finally, the results of another ANOVA analysis, graphically represented in Fig. 5, showed significant interaction between time of day and day of week also when trial types were collapsed (F(1,23) = 10.63, p < 0.001). Post hoc Bonferroni paired t-tests revealed a very strong difference in fP3As in the mornings, with Monday showing a shift to left asymmetry and Wednesday showing right asymmetry (*t*(23) = 15.35; *p* < 0.01) but no differences in the afternoons (*t*(23) = 1).

## 4 Discussion

The summary of the complete pattern of findings including the behavioral and ERP results is presented in Table 2.

**Table 2:** Summary of findings indicating RT (and accuracy criterion) differences between trial pair types, asymmetry, and time-dependent asymmetry.

Day	Time	RT (Accuracy)		Frontal P3 Asymmetry (Trial Type)	
				C	I
*Mon*	***AM***	iI = cC (>75%)	iC = cI (<75%)	Right	Right
	***PM***	iI < cC (>75%)	iC < cI (=75%)	None	Left
*Wed*	***AM***	iI > cC (>75%)	iC > cI (<75%)	Right	None
	***PM***	iI = cC (=75%)	iC = cI (=75%)	Right	None

**Note.***RT: Reaction time, C = Congruent, I = Incongruent. AM = morning; PM = afternoon. Accuracy criterion performance is 75%.*

The converging evidence pattern shown in Table 2, suggests that on Monday Morning the attentional resources, as reflected by right FP3As are allocated to equally select C or I and the effect of interference on RTs is annulled for pairs of same trials, but there seems to be the same approach for pairs of different trials associated with a significant drop in accuracy, suggesting no or poor retroactive switching. On Monday afternoon lack of FP3As show relatively effortful resource allocation for selecting C, for same trial pairs, which is associated with priming response to I trials and near-ceiling performance. In contrast, significant shift of FP3As to left lateralization indicates much more effortful control in selecting I for different trial pairs, as reflected by priming in response to C trials preceded by I trials. The latter suggests a retroactive switching effort to keep performance at a reasonably good level.

Wednesday morning and afternoon show the same patterns. For pairs of same trials, attentional resources, as reflected by right FP3As are allocated to select C, but for pairs of different trials lack of FP3As show relatively effortful resource allocation for selecting I. Although this pattern eliminates interference and keeps performance to a reasonably good level in the afternoon, and in the morning for same trial pairs, it does not seem to work as well for different trial pairs, whereby response to I is primed but performance is significantly worse, again suggesting inefficient retroactive switching.

These findings suggest that although retroactive switching during congruence judgment was affected at all testing times, it was most impaired in the mornings of both Monday and Wednesday, supporting a possible misalignment between circadian cycle and school start time, as we previously reported (D’Angiulli et al., 2021).

On Monday trials, the morning ERP activity associated with congruence judgment was generally frontal-right (F4) asymmetric for both congruent and incongruent trials, whereas in the afternoon the right activity decreased to same level as or significantly less than the one from the frontal-left (F3) region. The trend across every day and time combination shows more left fP3As amplitude during incongruent trials, this trend is significant only during the afternoon testing, but not the morning (see Fig. 5). This represents a key finding, as previous work theorizes that the ability to inhibit irrelevant information correlates with decrease in right-lateralized activity (Polich, 2012) and increased left-lateralized activity in the prefrontal regions (Ambrosini and Vallesi, 2017). In one study, transcutaneous direct current stimulation (tDCS) was applied to the dorsolateral prefrontal cortex (DLPFC) and resulted in improved reaction time during a Flanker Task, as well as increased P300 amplitude in the DLPFC region (Dubreuil-Vall et al., 2019). It was concluded that the ERP reflected tDCS-modulated changes in attention, conflict monitoring, and response inhibition. Taken together, we consider that P300 amplitude and hemispheric strength may provide a reliable measure of Stroop-like task response processing, including information inhibition and attention allocation. Taking this marker into account, it then appears that right-hemispheric P300 amplitudes were reduced during afternoon trials, and specifically during the most difficult incongruent trials. We provide two possible explanations for this pattern: 1) That circadian misalignment during early testing time can influence the participant's ability to perform on demanding conflict tasks, and this is reflected in a reduction in P300 amplitude in the right hemisphere, and 2) that incongruent trials recruit more frontal-right resources reflecting conflict management, context updating, and neural inhibition (Polich, 2007). Based on the present pattern of results, we put forth that both of these explanations are applicable, and that the frontal-right P300 amplitude reflects both external (i.e., time of day) and internal (i.e., cognitive load) factors.

The strongest differences between trials arose during the afternoon testing, supporting the idea that a combination of factors influences the P300 pattern shown here. Specifically, we show that morning testing produced non-significant trial-based P300 asymmetry differences regardless of the day, while both afternoon testing sessions produced larger differences in P300 amplitude lateralization between congruent and incongruent trials. Following Polich (2012)'s neuroinhibiton theory, we interpret this finding as a compensatory increased recruitment of frontal-right resources for incongruent stimulus evaluation, which may be reduced in morning testing or lack of sleep, as has been discussed previously (Deak and Stickgold, 2010). Our findings provide neural and behavioral evidence against early school starting and testing times, as they clearly demonstrate that attentional resource allocation may be reduced in the morning compared to the afternoon.

Taken together, the present findings illustrate that time of testing influences control of cognitive recruitment, an effect which is amplified when comparing congruent and incongruent trials. This therefore suggests that the combined influence P300 amplitude and frontal asymmetry are plausible correlates of cognitive function, and furthermore, the data implies that morning testing may be tapping less efficient recruitment of cognitive resources as reflected in decline of accuracy in congruence judgment performance. In the context of neuroeducation, we find these results to be preliminary but convincing evidence that the effects of early school start times appears to have objective measurable impact on cognitive and cortical processes. Indeed, it was shown that trials requiring more cognitive control (e.g., incongruent trials) were unsuccessful in recruitment of resources in the morning trials even when, on Monday morning, they were associated with a larger frontal-right P300 generally observed for “easy” tasks. However, this decrease in recruitment did not actually seem to reflect easier or more frequent conditions, since accuracy did not differ, and RTs did not favour incongruent trials. From an education standpoint, this evidence suggests that afternoon testing, or later school start times, would reduce effects of social jetlag on mental processes.

## 5 Limitations

The present study has some important limitations which we wish to explicitly highlight.

One aspect which we did not focus on during our analysis is the influence of individual chronotype on cognitive function. For instance, it is suggested that testing evening chronotypes in the morning can result in worse vigilance or executive function compared to individuals with a morning chronotype (Hicks et al., 2023). In another study it was found that evening chronotypes correlate negatively with academic achievement, while morning chronotypes show the opposite (Enright and Refinetti, 2017; Preckel et al., 2011). We also wish to acknowledge that our findings are limited to the computerized colour-word Stroop tasks, given that other conflict tasks (Stroop or Stroop-like) may probe different networks and recruit different regions depending on modality and response requirements (i.e., physical (computerized) or verbal).

Our previous work on this data focused on different correlates of cognition, and discusses further the effect of chronotype and social jetlag (D'Angiulli et al., 2023). However, our findings provide evidence of this jetlag effect without correction for chronotype, so it may be that categorizing individuals by chronotype may further highlight the effect of testing time.

Another important limitation was the lack of source analysis, which was impossible given the low number of electrodes, in order to proof that the asymmetry we observed was actually really linked to an underlying hemispheric asymmetry/lateralization. Other factors, such as dipole direction, may also contribute to the patterns presented here, as has been reported previously (Scherg et al., 2019). Still, the presently observed ERP asymmetry was specific to frontal electrodes and correlated with patterns in reaction times and accuracy (and other abovementioned aspects of adolescent functioning) which implies possible underlying asymmetry in task-related information processing. To really prove that our findings may correspond to phenotypic functional and structural hemispheric asymmetries, it would be crucial to follow up with a more rigorous replication study using high-density EEG/ERPs and source localization, specifically testing the neuroanatomical predictions of lateralization borne out of the neuroinhibition hypothesis.

## 6 Conclusions

In summary we found behavioral and neural evidence that switching during congruence judgment was affected at all testing times but most dramatically in the mornings of both early and mid school weeks, supporting misalignment between adolescent circadian cycle and school start time. We conclude by putting forth a preliminary interpretation that both time-of-day and trial type during a word-colour Stroop-like task are contributors to a novel combination of P300 amplitude and frontal hemispheric recruitment differences. We found that it appears that cognitive recruitment during testing in the morning is less efficient in terms of optimal accuracy performance and speed as compared to the afternoon, and that conflict (incongruence) amplifies this effect. Furthermore, we put forth the use of both P300 amplitude and asymmetry as a possible measure of cognitive control, allocation and resource recruitment that reflects ability in adolescents in real-world settings such as the classroom learning activities. This research, when taken alongside its complementary study investigating the behavioural aspects (D'Angiulli et al., 2023), may act as a starting point for studies investigating the effect of time-of-day on students' cognitive ability and may help to inform standardization, guidelines and best practices of school testing or ideal school timing during critical developmental periods such as puberty and adolescence.

## Funding

This research was funded by a Canada Research Chair award as well as seed funding from the Human Early Learning Partnership, 10.13039/501100005247University of British Columbia.

## CRediT authorship contribution statement

**Gabriel E. Byczynski:** Writing – review & editing, Visualization, Formal analysis. **Amedeo D'Angiulli:** Conceptualization, Data curation, Funding acquisition, Investigation, Methodology, Project administration, Resources, Supervision, Visualization, Writing – review & editing.

## Declaration of competing interest

The authors declare that they have no known competing financial interests or personal relationships that could have appeared to influence the work reported in this paper.

## Data Availability

Data will be made available on request.
