# Supplementary material for: Frontal P300 asymmetry and congruence judgment: Retroactive switching is impaired during school day mornings in female adolescents
Source: Curr Res Neurobiol. 2024 Mar 27;6:100128. doi: 10.1016/j.crneur.2024.100128 (PMC10990860; doi:10.1016/j.crneur.2024.100128)
Supplement: Multimedia component 1 [file mmc1.pdf]

Peer Review Overview

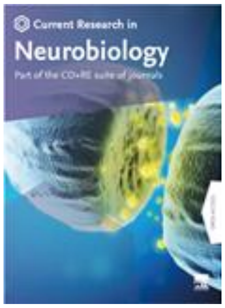

Manuscript Title: Frontal P300 asymmetry and congruence judgment: retroactive switching is impaired during school day mornings in female adolescents

Received	Feb 20, 2023
1st Decision	May 09, 2023
1st Revision Submitted	Jul 28, 2023
2nd Decision	Aug 18, 2023
2nd Revision Submitted	Dec 15, 2023
3rd Decision	Feb 03, 2024
3rd Revision Submitted	Feb 22, 2024
Accepted	Mar 07, 2024

1st Decision letter

Reference: CRNEUR-D-23-00015
Title: Frontal P300 asymmetry and congruence judgment: retroactive switching is impaired during school day mornings in female adolescents
Journal: Current Research in Neurobiology

Dear Mr Byczynski,

Thank you for submitting your manuscript to Current Research in Neurobiology.

I have completed my evaluation of your manuscript. The reviewers recommend reconsideration of your manuscript following major revision. I invite you to resubmit your manuscript after addressing the comments below. Please resubmit your revised manuscript by Jul 08, 2023.

When revising your manuscript, please consider all issues mentioned in the reviewers' comments carefully: please outline every change made in response to their comments and provide suitable rebuttals for any comments not addressed. Please note that your revised submission may need to be re-reviewed.

Current Research in Neurobiology values your contribution and I look forward to receiving your revised manuscript.

Kind regards,

Christopher I. Petkov
Editor in Chief
Current Research in Neurobiology

Comments from Editors and Reviewers:

Reviewer 1:

This is an interesting preliminary study on how scalp frontal hemispheric asymmetry of an important cognitive ERP component might represent a marker of cognitive control during task performance in interaction with modulations due to time of the day. I have however some comments on the current version of the manuscript.

1. Abstract: the authors state that "these findings suggest that the brain is able to act more efficiently during the afternoon". Why more efficiently? An efficient pattern would be for instance to find less activity to reach the same performance, but the authors find more (left-frontal) activity in the afternoon.
2. Abstract: something about behavioral performance data should be mentioned in the abstract.
3. What is the rationale for focusing on adolescent female population?
4. The authors did not consider participants' chronotypes (morning type vs. evening-type) in their sample. However this factor might explain a lot of interindividual variability, especially when one of the manipulations is the time of testing like in this study. Maybe this should be put among the limits in the discussion?
5. Congruent vs incongruent proportion manipulation was 1:3. When incongruent trials overcome congruent ones performance is facilitated as participants may proactively prepare for the more frequent incongruent stimuli (e.g., doi: 10.1016/j.cortex.2020.09.024). This should be commented and the choice of this proportion perhaps better explained.
6. Data is plural from Latin (please use this word as such and avoid for instance expressions like: "data was").
7. Discussion, page 17: "We interpret this however, as an increased reaction time that may actually reflect increased processing time of incongruent trials, which in turn reflects larger frontal-left P300 amplitudes". It is not clear how this interpretation is really different from the previously illustrated one (regarding decreasing performance), please clarify.
8. The authors interpret their results inferring that in some conditions there was more fatigue (e.g., page 18, last paragraph of the discussion before conclusions). However, fatigue should have been operationalized and measured more directly (and not just indirectly inferred) and used to corroborate explanations related to this factor. This should go in the limitation section of the discussion (now missing).

Reviewer 2:

Evidence for Frontal P300 Asymmetry as a Marker of Inhibitory Control and Cognitive Recruitment in Adolescent Females

The manuscript 'Evidence for Frontal P300 Asymmetry as a Marker of Inhibitory Control and Cognitive Recruitment in Adolescent Females' reports a single experiment aiming at assessing daily time's effect on adolescent cognitive control. Two groups of adolescents performed a Stroop task while recording frontocentral EEG on multiple days and times.
The study investigated a question of interest to a broad scientific community. Further specifying the ecological relevance of the cognitive control's neurophysiological correlates has the potential to match

the Current Research in Neurobiology audience.
However, the current version of the manuscript failed to provide sufficient background to motivate the study. The choice of the task seems at odd with both the previous investigations in the field and the aim of the study. Minimally this choice requires explicit justification from the authors. The reported results do not sufficiently inform the reader of the overall data pattern. This prevents the study from conveying convincing evidence supporting the conclusions.
Some suggestions are listed below for helping to improve the manuscript.

Introduction
-The authors reviewed previous investigations of ERP asymmetry across multiple cognitive domains. Still, only a little information is provided in the specific context of cognitive control or the P3 ERP. It would be more informative to focus this report on the processes investigated by the current study. Especially, the authors chose P3 lateralization as a cognitive control marker to explore its dynamic across multiple timing (day and time). The introduction needs to specify more explicitly what cognitive processing is deemed to be reflected by the P3 lateralization. Beyond the mentioned Go/No-Go or Oddball studies, multiple research investigated P3 ERP in the context of the Stroop task and are worth mentioning to provide some insights into the present study.
-Specifying the expected lateralized-P3 pattern in the context of the Stroop task, in relation to the previous comment, might help to further precise the hypotheses for the present study. Specifically, are the authors expecting more or less P3 lateralization due to Incongruency, Day, and Time?
-The authors mentioned that 'further research is necessary to establish reliable uses for asymmetry as a correlate of cognition'. On this basis, some readers might expect that the results section provide 1) a description of how the P3 lateralization reflects cognitive control in the Stroop task (e.g., accuracy, RT, interference score) and 2) the investigation of how this P3 pattern suggests that cognitive control is modulated across the timing conditions (day and time). The first stage is lacking in the current manuscript.

Method/Results
-The choice of the task is very specific and needs to be explicated. Classic Stroop task requires participants to respond to one stimulus feature (e.g., print color) while ignoring a second feature (e.g., written word) that can be congruent or incongruent. This setup evaluates the ability to control for/inhibit the interference generated by the incongruency (Stroop effect), which would align with the aims depicted in the introduction: investigating inhibition/cognitive control. In contrast, the task used in the present study required participants to detect whether the presented stimulus was congruent or incongruent. This modification of the paradigm is quite substantial and strongly impacts the engaged cognitive processes. The choice of this task version has to be justified regarding the authors' hypotheses. In addition, this new design of the Stroop task is combined with a 3:1 ratio in the number of incongruent vs. congruent trials. As the incongruency does not need to be processed but only detected to perform the task, it leads to a probabilistic bias in the response pattern, considerably reducing the engaged cognitive control. (If I always choose the 'incongruence' response, my accuracy is 75%)
-The number of trials on which rely the analyses need to be clarified. How many trials were performed by the participants? How many of these were rejected based on behavior or EEG preprocessing? It is mentioned that incorrect responses were not subject to further analysis. What was the success rate?
-Were the ERPs baseline corrected? This is especially important as the comparisons are performed

across subjects. Common ERP plots include the baseline time window.
-The number of participants also needs further clarification. From my uncertain understanding, 24 completed the study (12 mornings, 12 afternoons). 4 were discarded based on EEG or incomplete sessions. The correlation was performed on only 8 values. Could you clarify this and add degrees of freedom to the performed statistics (including for t and F tests) when not already included?
-'Both groups were tested on Monday and Wednesday of each week'. Was the experiment conducted longer than one week? If yes, were the data averaged across consecutive Mondays?
-The EEG setup, only 9 electrodes with an average reference, might lead to an increased signal-to-noise ratio that complicated the visual quantification of P3 amplitude. It would be informative to provide somewhere in the results some individuals' data visualization, e.g., scatter plot individual points in the P3 boxplots, allowing to appreciate directly within-group variability.
-Despite this short-communication manuscript focused on the P3 ERP, some basic behavioral data (e.g., accuracy, RTs, Congruency effect) would provide insight into the resulting EEG pattern.
-In Figure 1, averaging congruent and incongruent ERPs is noninformative regarding the P3 effect that differed according to congruency. Lateralized ERPs (differences between hemispheres) might allow for a clear display of the interaction between Asymmetry and the three investigated factors: Congruency, Day, and Time.
-Figure 1, when referring to ERPs, Power is less accurate than Amplitude or Voltage as an y label.
-If the Time factor is assessed through between-subject comparisons, the Congruency and Day factors can be assessed through within-subject comparisons.

Discussion
-To interpret the current data, it is proposed that P3 lateralization reflects response inhibition, in line with previous studies that actually investigated response inhibition. However, I still do not get how the present study investigated response inhibition (or any kind of inhibition).
-The main finding (larger left P3 lateralization on Monday afternoon) is described as 'counterintuitive'. It is associated with longer RT but interpreted as a better cognitive control recruitment (as compared to the morning group). Larger behavioral analyses, including accuracy and congruency effect, might clarify if the performance was poorer on Monday afternoon. If so, the left lateralization P3 might reflect a weaker cognitive control engagement. Alternatively, and more in line with the current authors' proposal, it could reflect more extensive recruitment of the cognitive control required by more conflictual processing (e.g., due to fatigue or circadian rhythms, as suggested by the authors). In any case, providing school-practice recommendations without aligning the behavioral and neurophysiological data appears premature.

1st Author Response Letter

Response to comments from Editors and Reviewers:

Comments from Reviewer 1:

This is an interesting preliminary study on how scalp frontal hemispheric asymmetry of an important cognitive ERP component might represent a marker of cognitive control during task performance in interaction with modulations due to time of the day. I have however some comments on the current version of the manuscript.
Thank you very much for your reviewer of our paper, please find below our response to each comment.

1. Abstract: the authors state that "these findings suggest that the brain is able to act more efficiently during the afternoon". Why more efficiently? An efficient pattern would be for instance to find less activity to reach the same performance, but the authors find more (leftfrontal) activity in the afternoon.
We thank the reviewer for this comment, however we believe that this statement was made in the press-release/media statement and not the abstract. Nonetheless, we will remove the term efficiency from the statement as it is not a current interpretation.

2. Abstract: something about behavioral performance data should be mentioned in the abstract.
We agree with the reviewer and have added behavioral data to the abstract, specifically relating the P300 amplitude with reaction times.

3. What is the rationale for focusing on adolescent female population?
We thank the reviewer for drawing this to our attention. Our rationale for a female population comes from studies showing that the risk associated with sleep deprivation, and the cognitive correlates of such, are more detrimental in females. We also base this decision on work showing that females typically require more sleep than males, and thus we can reduce inter-personal variability by restricting our sample to a single-sex. We have thus added these corrections to our paper to clarify our choice.

4. The authors did not consider participants' chronotypes (morning type vs. evening-type) in their sample. However this factor might explain a lot of interindividual variability, especially when one of the manipulations is the time of testing like in this study. Maybe this should be put among the limits in the discussion?
We agree with the reviewer's comment that chronotype has not been discussed explicitly in this paper. We have recently published another paper which looked at different data from this study, where we discuss further the possible effect of chronotype . We have included reference to this paper where necessary to better explain the data, and make reference to the chronotype we concluded based on the data presented there.

5. Congruent vs incongruent proportion manipulation was 1:3. When incongruent trials overcome congruent ones performance is facilitated as participants may proactively prepare for the more frequent incongruent stimuli (e.g., doi: 10.1016/j.cortex.2020.09.024). This should be commented and the choice of this proportion perhaps better explained.
We agree that this choice should be better explained and thus have added a section to the methods to clarify this choice, which itself was based on previous work using the same paradigm (now cited).

6. Data is plural from Latin (please use this word as such and avoid for instance expressions like: "data was").
We thank the reviewer for this attention to detail and have corrected our use of the term.

7. Discussion, page 17: "We interpret this however, as an increased reaction time that may actually reflect increased processing time of incongruent trials, which in turn reflects larger frontal-left P300 amplitudes". It is not clear how this interpretation is really different from the previously illustrated one (regarding decreasing performance), please clarify.
We agree with the reviewer that the added phrase is redundant in this instance, and have therefore removed it to improve clarity and readability.

8. The authors interpret their results inferring that in some conditions there was more fatigue (e.g., page 18, last paragraph of the discussion before conclusions). However, fatigue should have been operationalized and measured more directly (and not just indirectly inferred) and used to corroborate explanations related to this factor. This should go in the limitation section of the discussion (now missing).
We agree that fatigue should be an operationalized measure, and have thus included this as a limitation in the added section to the discussion.

Comments from Reviewer 2:

The manuscript 'Evidence for Frontal P300 Asymmetry as a Marker of Inhibitory Control and Cognitive Recruitment in Adolescent Females' reports a single experiment aiming at assessing daily time's effect on adolescent cognitive control. Two groups of adolescents performed a Stroop task while recording frontocentral EEG on multiple days and times.
The study investigated a question of interest to a broad scientific community. Further specifying the ecological relevance of the cognitive control's neurophysiological correlates has the potential to match the Current Research in Neurobiology audience.
However, the current version of the manuscript failed to provide sufficient background to motivate the study. The choice of the task seems at odd with both the previous investigations in the field and the aim of the study. Minimally this choice requires explicit justification from the authors. The reported results do not sufficiently inform the reader of the overall data pattern. This prevents the study from conveying convincing evidence supporting the conclusions.
Some suggestions are listed below for helping to improve the manuscript.
We thank the reviewer for the comments and feedback of our paper, and have provided an answer to each point below.
-The authors reviewed previous investigations of ERP asymmetry across multiple cognitive domains. Still, only a little information is provided in the specific context of cognitive control or the P3 ERP. It would be more informative to focus this report on the processes investigated by the current study. Especially, the authors chose P3 lateralization as a cognitive control marker to explore its dynamic across multiple timing (day and time). The introduction needs to specify more explicitly what cognitive processing is deemed to be reflected by the P3 lateralization. Beyond the mentioned Go/NoGo or Oddball studies, multiple research investigated P3 ERP in the context of the Stroop task and are worth mentioning to provide some insights into the present study.
We agree with the reviewer that more information regarding P300 ERP during the Stroop task would improve context for cognitive control. Accordingly, we have included works on this topic and discussed further which cognitive processes are being reflected. We also have framed our hypothesis to better

explain what process(es) we believe are reflected by P3 lat. (i.e., inhibitory ability and general resource recruitment).

-Specifying the expected lateralized-P3 pattern in the context of the Stroop task, in relation to the previous comment, might help to further precise the hypotheses for the present study. Specifically, are the authors expecting more or less P3 lateralization due to Incongruency, Day, and Time?
We agree with the reviewer and have added a more specific hypothesis as to which pattern is expected, with rational for why.

-The authors mentioned that 'further research is necessary to establish reliable uses for asymmetry as a correlate of cognition'. On this basis, some readers might expect that the results section provide 1) a description of how the P3 lateralization reflects cognitive control in the Stroop task (e.g., accuracy, RT, interference score) and 2) the investigation of how this P3 pattern suggests that cognitive control is modulated across the timing conditions (day and time). The first stage is lacking in the current manuscript.
We agree with the reviewer's comments and have added sections that discuss how P3 lateralization reflects control during the Stroop task, in order to strengthen the context and justification for the research.

Method/Results
-The choice of the task is very specific and needs to be explicated. Classic Stroop task requires participants to respond to one stimulus feature (e.g., print color) while ignoring a second feature (e.g., written word) that can be congruent or incongruent. This setup evaluates the ability to control for/inhibit the interference generated by the incongruency (Stroop effect), which would align with the aims depicted in the introduction: investigating inhibition/cognitive control. In contrast, the task used in the present study required participants to detect whether the presented stimulus was congruent or incongruent. This modification of the paradigm is quite substantial and strongly impacts the engaged cognitive processes. The choice of this task version has to be justified regarding the authors' hypotheses.
We agree with the reviewer that the task used in this study is quite a strong variation of the original Stroop task. We wish to assure the reviewer and reader that this was an intentional choice, and thus have provided clarification and justification for its use in the context of the research based on other previous work.

In addition, this new design of the Stroop task is combined with a 3:1 ratio in the number of incongruent vs. congruent trials. As the incongruency does not need to be processed but only detected to perform the task, it leads to a probabilistic bias in the response pattern, considerably reducing the engaged cognitive control. (If I always choose the 'incongruence' response, my accuracy is 75%)
We thank the reviewer for this comment. Our approach to measure accuracy separated congruent and incongruent trials. Accordingly, we did not see a biasing effect (i.e., near 100% for incongruent, and near 0% for congruent) as would be expected if the participant were always choosing incongruent. Furthermore, our participants were blinded to the ratio, preventing any predetermine strategy for answering. We do agree that these points should be clarified, and have included a statement in the methods highlighting this. We have also made reference to our complementary paper which focuses further on Stroop effect, accuracy, and a consideration for sleep.

-The number of trials on which rely the analyses need to be clarified. How many trials were performed by the participants? How many of these were rejected based on behavior or EEG preprocessing? It is mentioned that incorrect responses were not subject to further analysis. What was the success rate?

We thank the reviewer for drawing our attention to this missing information. We agree that it must be clarified, and have added the trial number, trial rejection, processing percent, and incorrect response usage to the methodology.

-Were the ERPs baseline corrected? This is especially important as the comparisons are performed across subjects. Common ERP plots include the baseline time window.
We agree that this is important information to include for an ERP study. We have added this information to the methodology section. We have extended the ERP plots to the onset of the trial, however because of the scale of the baseline period compared to the onset period, the two time points do not plot well on the same graph.

-The number of participants also needs further clarification. From my uncertain understanding, 24 completed the study (12 mornings, 12 afternoons). 4 were discarded based on EEG or incomplete sessions. The correlation was performed on only 8 values. Could you clarify this and add degrees of freedom to the performed statistics (including for t and F tests) when not already included?
We agree that clarification is required regarding the participant numbers and dof. As such, we have included better statements that are more explicit in stating the sample size, and dof for the statistic tests. The original number was 28 subjects, reduced to 24 after removing incomplete session or EEG. The correlation was performed only on the mean values of each condition, thus we plot 8 (trial type x time x day) conditions.

-'Both groups were tested on Monday and Wednesday of each week'. Was the experiment conducted longer than one week? If yes, were the data averaged across consecutive Mondays?
The data were averaged across consecutive days over a series of weeks. We agree that this should be clarified and have made the corresponding additions.

-The EEG setup, only 9 electrodes with an average reference, might lead to an increased signal-tonoise ratio that complicated the visual quantification of P3 amplitude. It would be informative to provide somewhere in the results some individuals' data visualization, e.g., scatter plot individual points in the P3 boxplots, allowing to appreciate directly within-group variability.
We thank the reviewer for this comment. Since our paper is mainly focused on central tendencies, we feel it may be confusing to include individual datapoints if they are not extensively discussed, a statement has been added to address this.

-Despite this short-communication manuscript focused on the P3 ERP, some basic behavioral data (e.g., accuracy, RTs, Congruency effect) would provide insight into the resulting EEG pattern.
We thank the reviewer for this note. In this instance we have included a statement in order to draw attention to the original paper, in which these are discussed in more detail.

-In Figure 1, averaging congruent and incongruent ERPs is noninformative regarding the P3 effect that differed according to congruency. Lateralized ERPs (differences between hemispheres) might allow for a clear display of the interaction between Asymmetry and the three investigated factors: Congruency, Day, and Time.
The averaged ERPs are intended to illustrate the effect of day and time on the P3 lateralization, however we agree that it does not illustrate the lateralization which occurs as a result of congruency. Figure 3 panel B was intended to suffice as illustrating the lateralization which occurs, however we agree that the ERP trace would add context. We have added a plot illustrating lateralized ERP for the congruent/incongruent effect.

-Figure 1, when referring to ERPs, Power is less accurate than Amplitude or Voltage as an y label.
We agree with this comment and have altered the y label to better represent the plotted data.

-If the Time factor is assessed through between-subject comparisons, the Congruency and Day factors can be assessed through within-subject comparisons.
We agree, and in line with the previous suggestions, have added plots which compare more directly the congruency and day factors through lateralization plots.

Discussion
-To interpret the current data, it is proposed that P3 lateralization reflects response inhibition, in line with previous studies that actually investigated response inhibition. However, I still do not get how the present study investigated response inhibition (or any kind of inhibition).
We thank the reviewer for this comment. We agree that our current manuscript requires strengthening of the argument in favour of inhibition. For the morning vs afternoon effect, and for the Monday vs Wednesday effect, in both cases our argument is that the differences in lateralization reflect either increased recruitment (or failure to increase recruitment). Since there is a 3:1 ratio of incongruent to congruent, even if there is continual pressing of the incongruent response, this response must be inhibited when the participant faces congruent stimuli, and vice-versa. Thus, while we agree that participant strategy may play a role, overall the effect remains that cognitive recruitment trends are reflective of increased (or impaired) cognition that reflects, in our opinion, inhibitory effects resulting from the (infrequent) stimulus type. Indeed, in the introduction we review studies that like ours consider Stroop-type effects as due to inhibition of contrasting responses or/and cognitive processing components (colour vs meaning). We have now included in the supplemental material, the results of individual condition accuracy and reaction time, including a sequence trial subdivision, which we believe supports our claim for inhibition, and, we believe, suggests against probabilistic biasing of responses. We do agree, however, that when considering AM vs PM lateralization, an increase in incongruent lateralization in the afternoon may reflect increased processing of the incongruent stimuli, which requires more assessment (reflected in Figure 2). Thus we will widen our interpretation beyond strictly inhibition, but also to cognitive recruitment.

-The main finding (larger left P3 lateralization on Monday afternoon) is described as 'counterintuitive'. It is associated with longer RT but interpreted as a better cognitive control recruitment (as compared to the morning group). Larger behavioral analyses, including accuracy and congruency effect, might clarify if the performance was poorer on Monday afternoon. If so, the left lateralization P3 might reflect a weaker cognitive control engagement. Alternatively, and more in line with the current authors' proposal, it could reflect more extensive recruitment of the cognitive control required by more conflictual processing (e.g., due to fatigue or circadian rhythms, as suggested by the authors). In any case, providing school-practice recommendations without aligning the behavioral and neurophysiological data appears premature.
We agree that the claims regarding school-practice may be outstepping the presented research alone, however we have aimed to justify our recommendations by making reference to the sister paper which is now properly mentioned. Together, we believe that the two papers provide sufficient evidence to warrant some recommendations in school practice, or at minimum a further consideration for this marker in an educational context. We have strengthened this section by justifying like above.

2nd Decision letter

Reference: CRNEUR-D-23-00015
Title: Frontal P300 asymmetry and congruence judgment: retroactive switching is impaired during school day mornings in female adolescents
Journal: Current Research in Neurobiology

Dear Mr Byczynski,

The reviewers have considered the revised manuscript and both advise a further review on points that I also feel need to be addressed. The reviewers recommend reconsideration of your manuscript following revision. I invite you to resubmit your manuscript after addressing the comments below. Please resubmit your revised manuscript by Oct 02, 2023.

When revising your manuscript, please consider all issues mentioned in the reviewers' comments carefully: please outline in a cover letter every change made in response to their comments and provide suitable rebuttals for any comments not addressed. Please note that your revised submission may need to be re-reviewed.

Current Research in Neurobiology values your contribution and I look forward to receiving your revised manuscript.

Kind regards,

Christopher I. Petkov
Editor in Chief
Current Research in Neurobiology

Comments from Editors and Reviewers:

Reviewer 1:

I have now revised the second version of this manuscript. I thank the authors for fully addressing some of my previous points. However, after reading the new version of the manuscript, the authors' response to previous comments, and the other reviewer's comments, I still have some concerns regarding a few pending (and new) points that I will list below.

Minor points
- Various statements in the manuscript are not fully justified by the data, in some cases because assumptions about the underlying constructs are implicitly made without directly testing them, in other cases because the statements are not fully compatible with the obtained data. Just a few examples follow:
o (Abstract) "morning testing impairs effective resource recruitment, as reflected in reduced P300 lateralization". Why does reduced P300 lateralization means less effective resource recruitment in this specific dataset? No evidence about this is provided.

o (Discussion) "We found reduced right lateralization of the P300 amplitude during incongruent trials, and during afternoon testing". When one looks at Table 1 concerning P300 at F3 and F4 for each condition, it is clear that during Wednesday afternoon P300 amplitude is actually numerically higher for F4 than F3 (with a statistically null effect for the incongruent condition, and a significant effect for the congruent condition). Therefore the statement is compatible only with results obtained in the Monday (not Wednesday) afternoon testing.
These above are just two examples of unwarranted statements.
- Another limitation that needs to be explicitly mentioned is that the P300 lateralization in the scalp cannot be confidently interpreted as a P300 lateralization in the brain (e.g., also midline brain generators might be expressed as lateralized patterns in the scalp, depending on the dipole orientation). However, source analysis is not performed to prove this hemispheric lateralization (which would be also impossible, given the low number of scalp electrodes employed = 9).
- An answer provided by the authors to one of my previous comments is: "We agree that this choice should be better explained and thus have added a section to the methods to clarify this choice, which itself was based on previous work using the same paradigm (now cited)". However, the manuscript does not cite any previous work concerning the modulation of cognitive control by the use of different ratios/proportions between congruent and incongruent conditions. Even worse, the authors state that: "Words were presented at a 3:1 ratio of incongruent (n=224) to congruent (n=76) stimuli. This was done in line with an 'Oddball Stroop' task, which results in a high incongruency demand, and thus high task control requirement". Actually a lot of classical and recent works (including the paper previously suggested and ignored, https://doi.org/10.1016/j.cortex.2020.09.024; also see for instance: https://doi.org/10.3758/BF03197535) argue the opposite, that is, lower cognitive control demands (or demands for other cognitive components: e.g., https://doi.org/10.5334/joc.232) for lower proportions of congruent vs. incongruent trials (an issue that has been somehow raised by both reviewers in the previous review round). This is also corroborated by the fact that, in the current (behavioral) dataset, accuracy was actually higher for incongruent vs. congruent trials, if I interpreted correctly the accuracy data reported in the Table 1 of the cited companion work by the same authors https://www.frontiersin.org/articles/10.3389/fpubh.2023.1022731/full#tab1. I believe that, for the readers' perusal, behavioral data should be also reported here and not only in the companion paper, if copyright issues allow this. In the light of this evidence, the authors should more clearly include as a limit the fact that their experimental paradigm is not optimal for taxing cognitive control.

Minor slip
- First page of introduction: "is literature that finds asymmetry to be". "There" should be added before "is" in this sentence.

Reviewer 2:

The authors addressed most of my remarks.

I am still not sure what cognitive processing the task they are using is targeting, as they mention 'attention', 'inhibitory control', and 'stimulus evaluation'. The paper would benefit from clarifying if these processes represent alternative hypotheses or multiple processes that the task engages altogether. In this regard, the justification of the (very uncommon) task design that was used is still partial and relies on citing a previous manuscript in which the justification was already partial. More specifically, I am still not convinced that the task engages inhibitory control. If this constitutes the main

hypothesis (as indicated by the title), this should be related to multiple previous investigations that coherently support it. Alternatively, this could be justified more extensively in the present manuscript.

2nd Author Response Letter

Response to comments from Editors and Reviewers:

Comments from Reviewer 1:

(Abstract) "morning testing impairs effective resource recruitment, as reflected in reduced P300 lateralization". Why does reduced P300 lateralization means less effective resource recruitment in this specific dataset? No evidence about this is provided.

- This claim is made on the basis that P300 amplitude changes, represented here, represent variation in resource recruitment. We discuss evidence for the relationship by noting the effect of circadian variation (via P300 amplitude changes) in resource recruitment. More generally though, we find resource recruitment to be an aspect of cognitive control, of which changes in P300 asymmetry have also been noted (Polich 2012). In order to further support these claims, we have added some extensive language in the introduction which discusses the role of the P300 in resource recruitment and cognitive control, in terms of inhibition and working memory. We agree that the effect may be ineffectively related to resource recruitment specifically, so our manuscript has been rephrased to discuss the effect of morning testing, for example, on the more general cognitive control (see 1.2 P300 and Cognitive Control, and 1.3 Frontal P300 Asymmetry).

(Discussion) "We found reduced right lateralization of the P300 amplitude during incongruent trials, and during afternoon testing". When one looks at Table 1 concerning P300 at F3 and F4 for each condition, it is clear that during Wednesday afternoon P300 amplitude is actually numerically higher for F4 than F3 (with a statistically null effect for the incongruent condition, and a significant effect for the congruent condition). Therefore, the statement is compatible only with results obtained in the Monday (not Wednesday) afternoon testing.

- Thank you for this note, we have addressed this inconsistency and reframed the results accordingly. Specifically the results now read: P300 amplitudes were significantly ($p < .05$) larger at the F4 electrode compared to F3 during both congruent and incongruent trials on Monday morning. On Monday afternoon, incongruent trials yielded significantly larger F3 P300 amplitudes compared to F4, whereas no asymmetry was observed for congruent trials. On Wednesday testing, for both morning and afternoon, congruent trials yielded a significantly larger P300 amplitude at the F4 electrode. However, there were no significant asymmetries for incongruent trials.

Reviewer 1 - General

Another limitation that needs to be explicitly mentioned is that the P300 lateralization in the scalp cannot be confidently interpreted as a P300 lateralization in the brain (e.g., also midline brain generators might be expressed as lateralized patterns in the scalp, depending on the dipole orientation). However, source analysis is not performed to prove this hemispheric lateralization (which would be also impossible, given the low number of scalp electrodes employed = 9).

- We agree that we cannot conclude lateralization from this data. Accordingly, the manuscript has been rephrased only to refer to asymmetry, describing only the physiological activity pattern, and not making unsubstantiated claims about lateralization at the structural neural level beyond electrode patterns.

An answer provided by the authors to one of my previous comments is: "We agree that this choice should be better explained and thus have added a section to the methods to clarify this choice, which itself was based on previous work using the same paradigm (now cited)". However, the manuscript does not cite any previous work concerning the modulation of cognitive control by the use of different ratios/proportions between congruent and incongruent conditions. Even worse, the authors state that: "Words were presented at a 3:1 ratio of incongruent (n=224) to congruent (n=76) stimuli. This was done in line with an 'Oddball Stroop' task, which results in a high incongruency demand, and thus high task control requirement". Actually a lot of classical and recent works (including the paper previously suggested and ignored, https://doi.org/10.1016/j.cortex.2020.09.024; also see for instance: https://doi.org/10.3758/BF03197535) argue the opposite, that is, lower cognitive control demands (or demands for other cognitive components: e.g., https://doi.org/10.5334/joc.232) for lower proportions of congruent vs. incongruent trials (an issue that has been somehow raised by both reviewers in the previous review round). This is also corroborated by the fact that, in the current (behavioral) dataset, accuracy was actually higher for incongruent vs. congruent trials, if I interpreted correctly the accuracy data reported in the Table 1 of the cited companion work by the same authors https://www.frontiersin.org/articles/10.3389/fpubh.2023.1022731/full#tab1. I believe that, for the readers' perusal, behavioral data should be also reported here and not only in the companion paper, if copyright issues allow this. In the light of this evidence, the authors should more clearly include as a limit the fact that their experimental paradigm is not optimal for taxing cognitive control.

- Thank you for this point. Firstly, we apologize for poorly explaining and justifying our approach. In order to directly address this, we have extended the introduction and methods sections to elaborate on the task choice, and what we believe is the most likely effect being observed (see paragraph 3 of section 1.2). This aspect also required additional results be presented which subdivided accuracy and reaction time by congruent and incongruent trial pairs, in line with other literature which is now cited. This dually addresses the concern about the availability of behavioural data, which we agree is a necessary addition for the clarity and context in this paper.

- Our perspective that our task does indeed test cognitive control to an extent, has also now been further discussed and supported in the manuscript. In brief, we have now included the extensive mention of response repetition and switching models, and the dual mechanism of control model, in conjunction with the task design, which we believe can support that our task, which now includes analyses at the trial level, investigated aspects of cognitive control including proactive (cognitive) control, inhibition, and reactive control.

- Confusion in this may arise, as the reviewer has noted literature which states that low PC results in lower cognitive control. In order to address this, we have aimed to clearly clarify the expected vs observed results at the behavioural level- which we believe illustrates both that 1) the reviewers are correct that a 75% incongruent may not be the most optimal task for probing control, but that 2) by taking into account the 75% marker as a 'low-cognitive control requirement threshold', our results can infer variation in cognitive control in this context by using the uncommon congruent trial as a probe for cognitive flexibility and switching. (Further discussed in the discussion and introduction).

Reviewer 1 – Minor slip

- First page of introduction: "is literature that finds asymmetry to be". "There" should be added before "is" in this sentence.

This section has been largely rewritten and no longer contains the error.

Comments from Reviewer 2:

I am still not sure what cognitive processing the task they are using is targeting, as they mention 'attention', 'inhibitory control', and 'stimulus evaluation'. The paper would benefit from clarifying if these processes represent alternative hypotheses or multiple processes that the task engages altogether. In this regard, the justification of the (very uncommon) task design that was used is still partial and relies on citing a previous manuscript in which the justification was already partial. More specifically, I am still not convinced that the task engages inhibitory control. If this constitutes the main hypothesis (as indicated by the title), this should be related to multiple previous investigations that coherently support it. Alternatively, this could be justified more extensively in the present manuscript.

- We agree with the reviewer that the previous manuscript was unclear as it related to many aspect of cognitive control without specifying their interactions. As will be clear in the extensive reformatting and writing, we actually strongly agreed with the reviewers wording of 'cognitive processes' and have instead focussed our paper on the more general cognitive control, which we believe more effectively captures the individual effects we have studied here. Indeed, the inter-dependency of the features of inhibition, attention, and evaluation mean that our paradigm does not optimally probe them individually, but rather captures the effect on general cognitive control. We have now included extensive discussion and support for our interpretation of cognitive control. Further, we believe that the switching aspect of cognitive control is the best representation of our findings, and indeed this model incorporates the processes previously discussed, but with better justification based on the available data.

- We have also now, following points raised by both reviewers, provided an extended justification of our task paradigm, and extended analyses which can be used to add context to our interpretation of cognitive control, and its inhibitory sub-domain. We have also altered our title, as an indirect interpretation of the reviewers comment, since we agree that the effects discussed in the manuscript are best captured with a more general and applicable term.

3rd Decision letter

Reference: CRNEUR-D-23-00015
Title: Frontal P300 asymmetry and congruence judgment: retroactive switching is impaired during school day mornings in female adolescents
Journal: Current Research in Neurobiology

Dear Mr Byczynski,

Thank you for submitting your manuscript to Current Research in Neurobiology.

We are able to tentatively accept your paper, pending one more minor revision from Reviewer 1, a

reconsideration of the lateralization issue and some clarity in the discussion on the limitations of the study.

I invite you to resubmit your manuscript after addressing the comments below. Please resubmit your revised manuscript in the next two weeks.

When revising your manuscript, please consider all issues mentioned in the reviewers' comments carefully: please outline every change made in response to their comments and provide suitable rebuttals for any comments not addressed. Please note that your revised submission may need to be re-reviewed.

Current Research in Neurobiology values your contribution and I look forward to receiving your revised manuscript.

Kind regards,

Christopher I. Petkov
Editor in Chief
Current Research in Neurobiology

Comments from Editors and Reviewers:

Reviewer 1:

The authors addressed most of my previous comments. I do not agree that the asymmetry issue was solved by just avoiding talking about lateralization. The authors keep explicitly referring to asymmetries (a limitation section should more explicitly mention the lack of source analysis, which is impossible given the low number of channels, to proof that this asymmetry is actually really linked to an underlying hemispheric asymmetry/lateralization). Please also note that some authors' names were accidentally cut from the reference list.

3rd Author Response Letter

Response to comments from Editors and Reviewers:

Comments from Reviewer 1:

1. I do not agree that the asymmetry issue was solved by just avoiding talking about lateralization. The authors keep explicitly referring to asymmetries (a limitation section should more explicitly mention the lack of source analysis, which is impossible given the low number of channels, to proof that this asymmetry is actually really linked to an underlying hemispheric asymmetry/lateralization).

We thank the reviewer for this additional comment on the discussion of lateralization. We fully agree with the reviewer that we cannot definitively conclude that asymmetry is linked with hemispheric asymmetry at the neural level without a source analysis, which we concur is not possible with the number of electrodes.

We have now included a section of limitations with a more explicit statement which indicates that without source analysis, it is not possible to conclusively state that our sensor-level analysis is representative of the underlying hemispheric asymmetry, whether functional or structural (whose links are currently hotly debated in neuroscience, see special issue on Sperry in Brain Structure and Function journal, 2021). We have also included an additional statement of clarification regarding how we interpret the asymmetry indicated by our findings, and we have suggested that possible underlying genuine hemispheric asymmetry should be more rigorously tested in further multi-methods studies.

2.Please also note that some authors' names were accidentally cut from the reference list.

We have made the corresponding corrections, and thank the reviewer for their thorough comments which have undoubtedly improved the quality of the manuscript.

Accept Letter

Dear Mr Byczynski,

Thank you for entrusting the review of your manuscript to Current Research in Neurobiology. I do apologize if the process was overly long but am pleased to inform you that your manuscript has been accepted for publication.

Your accepted manuscript will now be transferred to our production department. We will create a proof which you will be asked to check, and you will also be asked to complete a number of online forms required for publication. If we need additional information from you during the production process, we will contact you directly.

We appreciate and value your contribution to Current Research in Neurobiology. We regularly invite authors of recently published manuscript to participate in the peer review process. If you were not already part of the journal's reviewer pool, you have now been added to it. We look forward to your continued participation in our journal, and we hope you will consider us again for future submissions.

CRNEUR aims to be a unique, community-led journal, as highlighted in the Editorial Introduction. As part of this vision, we will be regularly seeking input from the scientific community and encourage you and your co-authors to take the survey.

We would also like to invite you to take part in our CRNEUR Author Question & Answer (Q&A), which could get published alongside your article and help to promote it. We suspect you might have an interesting story of perseverance or team work that was required for the research study to complete, or a diversity of perspectives that you might share, as a way of inspiring others about neuroscience.

Kind regards,

Christopher I. Petkov
Editor in Chief
Current Research in Neurobiology

-------- *End of Review Comments* --------
